# Indications and complications for surgical management of thyroid diseases: A single center experience

**DOI:** 10.1016/j.amsu.2022.103980

**Published:** 2022-06-14

**Authors:** Khalid A. Alyahya, Abdullah A. Alarfaj, Abdulwahab A. Alyahya, Abdulrahman E. Alnaim

**Affiliations:** a Head & Neck Surgery College of Medicine, King Faisal University, Al-Hasa, Saudi Arabia; b College of Medicine, King Faisal University, Al-Hasa, Saudi Arabia

## Abstract

**Background:**

Thyroid disorders are one of the most common endocrine disorders. Thyroid disorders are highly prevalent in the Saudi population. There are many approaches to treat thyroid disorders, varying from conservative to surgical, depending on the severity of each condition. There are many indications for surgical management of thyroid diseases, including carcinoma, hyperthyroidism, and local compression symptoms.

**Materials and methods:**

This is a retrospective study included subjects operated upon for a thyroid disorder over 6 years period in the eastern province of Saudi Arabia.

**Results:**

The clinical manifestations and postoperative characteristics of the patients are given in Table 2. It can be observed that the most dominant clinical presentation was neck mass (76.4%), while the most common indication of surgery was the suspicion of cancer (54.5%). Furthermore, the most common histopathology was papillary thyroid cancer (41.8%), whereas the most common complication after the surgery was hypocalcemia (9.1%). Likewise, total thyroidectomy was the most commonly sought surgical intervention (70.9%). Additionally, Bethesda class III constitutes 35.1%, while Bethesda class II constitutes 29.7%.

**Conclusion:**

Suspicion of cancer was the most prominent indication for surgical intervention. The most prevalent postoperative complications were hypocalcemia. Furthermore, papillary thyroid carcinoma was the most common histological findings, which raises several questions since it contrasts with previous studies done in Saudi Arabia. We believe that the number of thyroid procedures in our region is underreported, and more research is needed to validate this.

## Highlights

• Most common indication for thyroid surgery was suspicion of malignancy.
• Most common post-operative histopathological finding was papillary thyroid cancer.
• Most common surgery done was total thyroidectomy.
• Majority of patient presented clinically with neck mass.
• Hypocalcemia was the most common post-operative complication observed.
• There's a lack of published research regarding the same topic.

## 1 Introduction

Thyroid disorders are one of the most common endocrine disorders [1]. According to the World Health Organization (WHO), more than 190 million people have iodine deficiency disorders [2]. Thyroid gland is essential for the human body and it's important for body metabolism. Dysfunction of the thyroid gland has a major effect on human health and quality of life [3].

Thyroid disorders are highly prevalent in Saudi population [4]. Thyroid malignancy is the second most common malignancy affecting women between the age of 35–39 years and the eighth among males in Saudi Arabia [5]. According to the 2015 Saudi Cancer Registry report, there were a total of 1020 thyroid cases cancer accounting to 8.5% from all newly diagnosed cancers in 2015. Also, thyroid cancer affected 793 (77.7%) females and 227 (22.3%) males; with a female to male ratio of 3.5:1.

There are many approaches to treat thyroid disorders, varying from conservative to surgical, depending on the severity of each condition. Surgical management of thyroid disorders is a common procedure all over the world [6]. In Germany, the number of thyroid surgeries between 2005 and 2013 was 79,000 to 89,000 per year [7]. Furthermore, in many cases, thyroid surgery remains the treatment of choice, despite the advances of conservative management [8].

There are many indications for surgical management of thyroid diseases, including carcinoma, hyperthyroidism, and local compression symptoms [9]. A recent study done in Saudi Arabia on 129 cases shows that the most common indication for surgical management is local compression symptoms, which accounts for 43% of all cases [8]. Furthermore, out of 129 cases, 9 patients had Recurrent laryngeal nerve injury (RLN) (8). Moreover, RLN is considered to be the most common and serious surgical complication relative to other complications such as hematoma and hypocalcemia [8].

There are a limited number of studies in Saudi Arabia that point out the surgical indication of thyroid disorders in Eastern province, particularly in Al-Ahsa city. Hence, this study aims to highlight the surgical indication for thyroid disorders and to evaluate the rate and type of postoperative complications in Eastern province, Al-Ahsa city.

## 2 Materials and methods

This is a retrospective study included subjects operated upon for a thyroid disorder over 6 years period in the eastern province of Saudi Arabia from January 2015 to December 2020. All patients who had any thyroid disorder and underwent thyroid surgery were included in this study. Patients who were not diagnosed with any thyroid disorder or did not undergo any thyroid surgery were excluded from this study. Patient data was collected from National guard hospital record in Al-Ahsa city. The data was used to gather information on the following variables: socio-demographics, the clinical diagnosis, the manifestation of thyroid lesions, Indication of thyroid Surgery, histopathology pattern of thyroid lesion, and post-operative complications. The patient's personal information kept confidential with complete privacy. The study was approved by the Ethics Research Committee at the College of Medicine, King Faisal University.

Quantitative data are presented using mean ± Standard deviation (SD) or median with interquartile range if appropriate. Qualitative data are presented using counts and proportions (%). Between comparisons, Fischer Exact test (categorical variables) and paired sample *t*-test (continuous variables) were applied. A p-value of ≤0.05 (two-sided) was used to indicate statistical significance. All data analyses were performed using the Statistical Packages for Social Sciences (SPSS) version 26 Armonk, NY: IBM Corp.

This study has been reported in line with the STROCSS criteria [20].

## 3 Results

We analyzed 55 patients to determine the indications and complications for surgical management of thyroid diseases. As seen in Table 1, the mean age of the patients was 45.9 (SD 13.9) years old with females dominating the males (87.3% vs 12.7%). Nearly all (90.9%) were Al Ahsa residents compromising with mostly Saudis (94.5%). Furthermore, 81.8% were admitted to general surgery. The most common diagnosis related to thyroid disease was thyroid nodule (65.5%).

**Table 1:** Baseline characteristics of patients ^(n = 55)^.

Study variables	N (%)
Age in years (mean ± SD)	45.9 ± 13.9
Gender	
• Male	07 (12.7%)
• Female	48 (87.3%)
Place of residence	
• Al Ahsa	50 (90.9%)
• Non-Al Ahsa	05 (09.1%)
Nationality	
• Saudi	52 (94.5%)
• Non-Saudi	03 (05.5%)
Department	
• General surgery	45 (81.8%)
• ENT	10 (18.2%)
Diagnosis of thyroid a	
• Thyroid nodule	36 (65.5%)
• Thyroid cancer	19 (34.5%)

a Non-thyroid cases were excluded from the analysis.

**Table 2:** Clinical manifestations and Postoperative characteristics of patients ^(n = 55)^.

Variables	N (%)
Clinical presentation	
• Neck mass without pressure symptoms.	42 (76.4%)
• Neck Mass with pressure symptoms.	12 (21.8%)
• Others	01 (01.8%)
Indication of surgery	
• Suspicion of cancer	30 (54.5%)
• Compression symptoms	08 (14.5%)
• Thyroid nodules	09 (16.4%)
• Papillary thyroid cancer	03 (05.5%)
• Goiter	03 (05.5%)
• Others	02 (03.6%)
Histopathology	
• Papillary thyroid cancer	23 (41.8%)
• Multinodular colloid goiter	19 (34.5%)
• Follicular adenoma	04 (07.3%)
• Hyperplastic nodule	02 (03.6%)
• Thyroid nodule	04 (07.3%)
• Others	03 (05.5%)
Postop complication	
• None	41 (74.5%)
• Hypocalcemia	05 (09.1%)
• Right vocal cord paralysis	02 (03.6%)
• RLN injury	02 (03.6%)
• Others	05 (09.1%)
Type of surgery a	
• Total thyroidectomy	39 (70.9%)
• Hemithyroidectomy/Lobectomy	16 (29.1%)
Bethesda classification	
• None	03 (05.5%)
• I	0
• II	16 (30.8%)
• III	21 (40.4%)
• IV	02 (03.8%)
• V	10 (08.2%)
• VI	03 (05.5%)

a Patients who underwent parathyroidectomy were excluded from the analysis.

The clinical manifestations and postoperative characteristics of the patients were given in Table 2. It can be observed that the most dominant clinical presentation was neck mass (76.4%) while the most common indication of surgery was the suspicion of cancer (54.5%). The most common histopathology was papillary thyroid cancer (41.8%) whereas the most common complication after the surgery was hypocalcemia (9.1%). Total thyroidectomy was the most commonly sought surgical intervention (70.9%). Bethesda class III constitutes 40.4% while Bethesda class II constitutes 30.8%.

When measuring the differences of laboratory parameters before and after surgery, we observed that the mean value of PTH was statistically significantly lower postoperatively (mean diff. = -2.809; p = 0.003) while the differences of TSH, T4 and Ca before and after surgery were not statistically significant (p > 0.05) (see Table 3).

**Table 3:** Paired T-test of the laboratory parameters pre and post-surgery ^(n = 55)^.

Variable	Post Mean ± SD	Pre Mean ± SD	Mean Diff.	95% CI	*t*-test	P-value^§^
TSH	6.19 ± 11.9	8.14 ± 12.5	−1.948	−6.921–3.025	−0.788	0.435
T4	12.8 ± 22.9	13.9 ± 20.8	−1.052	−11.938–9.864	−0.196	0.846
Ca	5.20 ± 8.85	6.67 ± 5.43	−1.464	−4.459–1.532	−0.985	0.330
PTH	3.98 ± 2.69	6.78 ± 4.76	−2.809	−4.595–1.023	−3.212	0.003 **

^§^ P-value has been calculated using paired sample *t*-test.

** Significant at p < 0.05 level.

When measuring the relationship between the type of the surgery in regards to the clinical manifestations and postoperative characteristics of the patients, it was found that the prevalence of patients who had a suspicion of cancer as an indication of surgery (p = 0.021) and papillary thyroid cancer as diagnosis during histopathology (p = 0.001) was more common among those who underwent total thyroidectomy while clinical presentation, postop complication, and Bethesda classification did not show a significant relationship in both thyroidectomy and lobectomy surgical procedures (p > 0.05) (see Table 4).

**Table 4:** Association between the type of the surgery among Clinical manifestations and Postoperative characteristics of patients ^(n = 55^^)^.

Factor	Thyroidectomy N (%) ^(n = 39)^	Lobectomy N (%) ^(n = 16)^	P-value^§^
Clinical presentation			
• Neck mass without pressure symptoms	32 (82.1%)	10 (62.5%)	0.101
• Neck Mass with pressure symptoms	07 (17.9%)	05 (31.3%)	
• Others	0	01 (06.3%)	
Indication of surgery			
• Suspicion of cancer	26 (66.7%)	04 (25.0%)	0.021 **
• Compression symptoms	04 (10.3%)	04 (25.0%)	
• Thyroid nodules	05 (12.8%)	04 (25.0%)	
• Papillary thyroid cancer	02 (05.1%)	01 (06.3%)	
• Goiter	02 (05.1%)	01 (06.3%)	
• Others	0	02 (12.5%)	
Histopathology			
• Papillary thyroid cancer	21 (53.8%)	02 (12.5%)	0.001 **
• Multinodular colloid goiter	14 (35.9%)	05 (31.3%)	
• Follicular adenoma	01 (02.6%)	03 (18.8%)	
• Hyperplastic nodule	01 (02.6%)	01 (06.3%)	
• Thyroid nodule	0	04 (25.0%)	
• Others	02 (05.1%)	01 (06.3%)	
Postop complication			
• None	29 (74.4%)	12 (75.0%)	0.852
• Hypocalcemia	04 (10.3%)	01 (06.3%)	
• Right vocal cord paralysis	01 (02.6%)	01 (06.3%)	
• RLN injury	01 (02.6%)	01 (06.3%)	
• Others	04 (10.3%)	01 (06.3%)	
Bethesda classification a			
• II	11 (29.7%)	05 (33.3%)	0.133
• III	13 (35.1%)	08 (53.3%)	
• IV	01 (02.7%)	01 (06.7%)	
• V	10 (27.0%)	0	
• VI	02 (05.4%)	01 (06.7%)	

^§^ P-value has been calculated using Fischer Exact test.

** Significant at p < 0.05 level.

a None cases were excluded from the analysis.

## 4 Discussion

Thyroid disorders are one of the most common endocrine disorders [1]. Thyroid disorders are highly prevalent in Saudi population [4]. There are many indications for surgical management of thyroid diseases, including carcinoma, hyperthyroidism, and local compression symptoms [6]. This study was conducted retrospectively from January 2015 to December 2020 which included all patients with a thyroid disorder and underwent surgical treatment.

Among 55 patients included in the current study, it can be observed that the most dominant clinical presentation was neck mass (98.2%), while the most common indication of surgery was the suspicion of cancer (54.5%). Furthermore, the most common histopathology was papillary thyroid cancer (41.8%), whereas the most common complication after the surgery was hypocalcemia (9.1%). Likewise, total thyroidectomy was the most commonly sought surgical intervention (70.1%). Additionally, Bethesda class III constitutes 40.4% while Bethesda class II constitutes 30.8%.

Female gender is always more prevalent in thyroid disorders as 87.3% of the studied sample were females, which is, in fact, consistent with prior published articles [8,5]. Furthermore, young patients tend to be diagnosed with thyroid disorders more than elderly as the mean age in the current study was 46 years, which is consistent with three studies done in Saudi Arabia [10,11,8]. For the type of surgery that was preformed, total thyroidectomy was the most commonly sought surgical intervention which is similar to what was found in Milan D. Jovanovic et al. study [12].

Neck mass was the most common presenting clinical manifestation in the studied population (98.2%), which corresponds with the Ali M. et al. study [13]. On contrary, according to Alshareef B. et al. study the most common clinical manifestation was local compression symptoms [8].

As part of histopathology, the current study reveals a unique finding in which the majority of cases had papillary thyroid carcinoma, which contradicts several studies done inside and outside Saudi Arabia. According to Milan D. Jovanovic et al., most of the cases had benign thyroid disorders [12]. Furthermore, In Ashok K. et al. study more than (90%) of the analyzed cases had benign histopathological findings [14].

There are various indications for surgical intervention in thyroid diseases, ranging from suspicion of cancer to cosmetic purposes. As demonstrated in the current study, suspicion of cancer was the leading indication for surgical intervention. In comparison with previous articles, Bhattacharyya N. et al. study found that the most common indication was suspicion of cancer [15]. Furthermore, a systematic review conducted in Germany among 2043 cases, showed findings that are consistent with the current study [16]. On contrary, a study conducted in the western region of Saudi Arabia found that the most common indication was local compression symptom, which is considered the third most common indication after suspicion of cancer [8]. Additionally, according to Alkhars A. et al. study, thyroid nodule is the most common indication for surgical intervention, which contradicts the current study findings [17].

Surgical intervention in thyroid diseases has some risks and complications. As per complications hypocalcemia was the most common complication in the current study. Regarding laboratory findings, it was found that the mean Postoperative PTH level was significantly lower compared to the pre-operative PTH measurement. According to P. Stathopoulos et al., the most common surgical complication was transient hypoparathyroidism [18]. Additionally, as per Bellantone R. et al. hypocalcemia was the most common complication for surgical intervention which is in agreement with current study [19]. Nevertheless, Alshareef B. et al. found that recurrent laryngeal nerve injury was the most common complication postoperatively [8].

## 5 Conclusion

The current study findings strongly suggest that thyroid diseases are more frequent in women than in men. Suspicion of cancer was the most prominent indication for surgical intervention. The most prevalent post-operative complications were hypocalcemia. Furthermore, papillary thyroid carcinoma was the most common histological findings, which raises several questions since it contrasts with previous studies done in Saudi Arabia. We believe that the number of thyroid procedures in our region is underreported, and more research is needed to validate this.

## Ethical approval

The study was approved by King Faisal University College of Medicine's Ethics Research Committee with references number (13 - 10 - 2020), and all requirement document will be provided when needed.

## Sources of funding

The authors appreciate and acknowledge King Faisal University for their support, as this article is entirely funded by 10.13039/501100004686King Faisal University's Deanship of Scientific Research. Also, we declare that Deanship of Scientific Research is not involved in the collection, analysis and interpretation of data; in the writing of the manuscript; and in the decision to submit the manuscript for publication, and all were the authors role and decision.

## Author contribution

Khalid A. Alyahya: principle investigator, contribute in study concept, and writing the paper, Abdullah A. Alarfaj: co-author, contribute in study concept, and writing the paper, Abdulwahab A. Alyahya: co-author, contribute in study concept and design, data collection, data analysis and interpretation, writing the paper. Abdulrahman E. Alnaim: co-author and corresponding author, contribute in data collection, data analysis and interpretation, writing the paper.

## Registration of research studies

Name of the registry: research registry.

Unique Identifying number or registration ID: researchregistry7402.

Hyperlink to your specific registration (must be publicly accessible and will be checked): https://researchregistry.knack.com/research-registry#user-researchregistry/registerresearchdetails/619fb34ad839e9001fd6ba90/

## Guarantor

Authors are the guarantors of this article and they have the full responsibility for the work.

## Declaration of competing interest

All authors declare that they have no conflicts of interest, and that they have no financial or personal relationships with any people or organizations that could improperly affect (bias) our work.
